# Designing Epigenetic Clocks for Wildlife Research

**DOI:** 10.1111/1755-0998.14120

**Published:** 2025-05-06

**Authors:** Levi Newediuk, Evan S. Richardson, Alyssa M. Bohart, Amélie Roberto‐Charron, Colin J. Garroway, Meaghan J. Jones

**Affiliations:** ^1^ Department of Biological Sciences University of Manitoba Winnipeg Manitoba Canada; ^2^ Environment and Climate Change Canada Winnipeg Manitoba Canada; ^3^ Department of Environment Government of Nunavut Iqaluit Nunavut Canada; ^4^ Department of Biochemistry and Medical Genetics University of Manitoba Children's Hospital Research Institute of Manitoba Winnipeg Manitoba Canada

**Keywords:** age estimation, biodiversity conservation, biomarker, DNA methylation, epigenetic clock, wildlife monitoring

## Abstract

The applications of epigenetic clocks – statistical models that predict an individual's age based on DNA methylation patterns – are expanding in wildlife conservation and management. This growing interest highlights the need for field‐specific design best practices. Here, we provide recommendations for two main applications of wildlife epigenetic clocks: estimating the unknown ages of individuals and assessing their biological ageing rates. Epigenetic clocks were originally developed to measure biological ageing rates of human tissues, which presents challenges for their adoption in wildlife research. Most notably, the estimated chronological ages of sampled wildlife can be unreliable, and sampling restrictions limit the number and variety of tissues with which epigenetic clocks can be constructed, reducing their accuracy. To address these challenges, we present a detailed workflow for designing, validating applying accurate wildlife epigenetic clocks. Using simulations and analyses applied to an extensive polar bear dataset from across the Canadian Arctic, we demonstrate that accurate epigenetic clocks for wildlife can be constructed and validated using a limited number of samples, accommodating projects with small budgets and sampling constraints. The concerns we address are critical for clock design, whether researchers or third‐party service providers perform the bioinformatics. With our workflow and examples, we hope to support the accessible and widespread use of epigenetic clocks in wildlife conservation and management.

## 1 Introduction

Over the past decade, epigenetic clocks – statistical models that predict age based on DNA methylation patterns – have transformed human biomedicine by revealing how stressful life experiences accelerate biological ageing, which is associated with disease (Lu et al. 2019) and early mortality (Chen et al. 2016; Marioni et al. 2015). Now, epigenetic clocks are poised for similar impacts in wildlife management and conservation biology. Biological age, estimated by epigenetic clocks, provides a non‐lethal means to estimate key metrics for conservation and management, including age structure and cumulative lifetime stress, which underlie individual survival and population declines. Although other biological ageing methods have provided some of these insights, the superior precision and accuracy of epigenetic clocks set them apart as a uniquely promising tool (Le Clercq et al. 2023).

While a universal clock was recently published for all mammals (Lu et al. 2023), the most accurate epigenetic clocks are species‐specific. Custom clocks present a new design challenge. Relative to sample collection from humans and model organisms, which happens in highly controlled settings, wildlife sampling is logistically challenging, often underfunded and time‐intensive, making it difficult to choose who and what is sampled. The small sample sizes typical of wildlife studies can be biased towards specific tissue types, sexes and ages – all variables associated with distinct DNA methylation patterns (McEwen et al. 2020; Simpkin et al. 2016; Yusipov et al. 2020). The chronological ages of individuals used to train epigenetic clocks are often estimated and thus imprecise, contributing to errors that make clock predictions less accurate. Several species‐specific epigenetic clocks have already been developed (Bors et al. 2021; Czajka et al. 2024; Newediuk et al. 2024; Parsons et al. 2023); however, there has been limited discussion on best practices for sampling wildlife DNA and designing epigenetic clocks to deal with biases and ageing error. Few species‐specific clocks have been independently validated for accuracy across studies, making it difficult to detect when biases are present. Critically, third‐party services now enable researchers with minimal bioinformatic and epigenetic clock experience to outsource the development of epigenetic clocks. An understanding of the clock‐building process and sources of sampling bias is essential for those building or using wildlife epigenetic clocks, whether the work is done in‐house or by a third‐party service.

**FIGURE 1:**
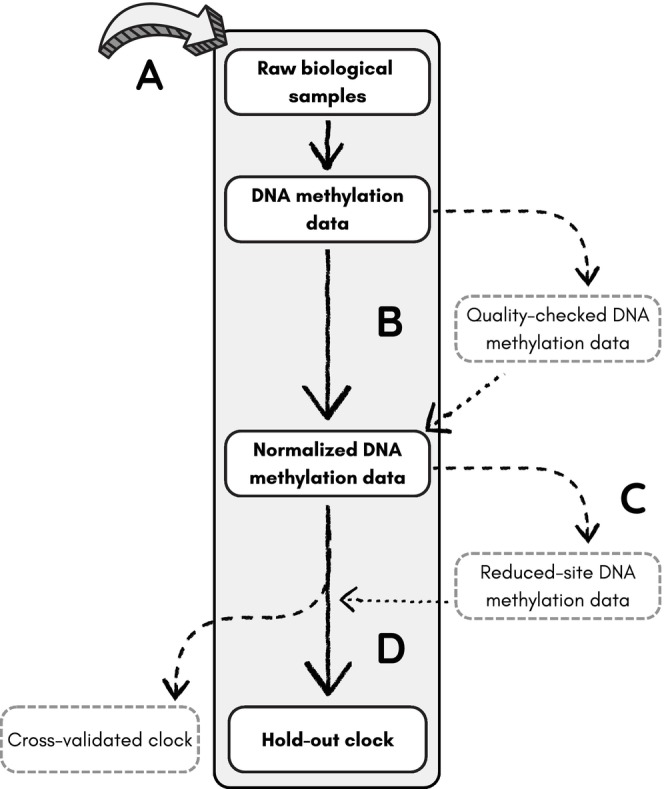
Our recommended workflow (solid lines) for developing epigenetic clocks includes (A) deciding on the sample size and characteristics required to train an accurate clock, extracting the DNA and quantifying DNA methylation; (B) performing optional quality‐control tests and normalising the DNA methylation data; (C) performing pre‐processing steps to limit the number of features used to fit the clock and (D) validating the clock. Dotted lines indicate optional or alternative steps.

This paper offers practical recommendations for designing species‐specific epigenetic clocks for wildlife, with a focus on minimising the effects of sampling bias on their accuracy. We begin with an overview of epigenetic clock models, covering what they measure and their potential applications in wildlife conservation and management. Then, we discuss the key design considerations important for minimising bias in wildlife epigenetic clocks, including representative sampling, feature selection and validation methods sensitive to small sample sizes. We frame our discussion around comparisons of epigenetic clock design approaches using simulations and an extensive DNA methylation dataset from several wild polar bear ( *Ursus maritimus* ) populations (Box 1). Accompanying the discussion, we provide a comprehensive workflow that guides the reader through each major step and decision in developing a species‐specific epigenetic clock (Figure 1).

BOX 1 **Polar Bear Data From Across the Canadian Arctic.**

We compiled an extensive DNA methylation dataset from polar bears sampled across the Canadian Arctic to assess whether sampling biases, data pre‐processing, and validation influence wildlife clock performance. Our dataset is comprised of polar bear DNA sampled from 10 distinct subpopulations, each with different proportions of blood, skin muscle tissue. Samples are from male and female bears and represent ages across the typical lifespan of a wild polar bear from age 0 to 30 (Table B1).

**TABLE B1:** Overview of polar bear DNA methylation samples from 10 genetically distinct subpopulations across the Canadian Arctic. DNA was extracted from three tissue types: Blood (B), skin (S) and muscle, and male (M) and female (F) bears.

Subpopulation	Number of samples	Location	Age range	Tissue proportions	Sex proportions
Southern Beaufort	76	Western Arctic	0–20	B: 0.20; S: 0.80	F: 0.54; M: 0.46
Northern Beaufort	62	Western Arctic	0–24	B: 0.11; S: 0.89	F: 0.55; M: 0.45
Gulf of Boothia	36	Western Arctic	0–20	M: 1.0	F: 0.53; M: 0.47
Lancaster Sound	41	Western Arctic	0–21	M: 1.0	F: 0.46; M: 0.54
Mc'Clintock Channel	35	Western Arctic	0–17	M: 1.0	F: 0.66; M: 0.34
Foxe Basin	40	Central Arctic	0–21	M: 1.0	F: 0.50; M: 0.50
Western Hudson Bay	235	Central Arctic	0–30	B: 0.43; S: 0.57	F: 0.60; M: 0.50
Southern Hudson Bay	47	Central Arctic	0–22	M: 1.0	F: 0.51; M: 0.49
Davis Strait	41	Eastern Arctic	0–20	M: 1.0	F: 0.46; M: 0.54
Baffin Bay	40	Eastern Arctic	0–23	M: 1.0	F: 0.50; M: 0.50

We used the age, tissue, sex and population structure of the data to evaluate the effects of class bias, age bias and feature selection on clock performance. We trained clocks using varying degrees of overlap (0%–100%) between the age ranges, tissues, sexes and populations in the training and testing sets. We fit these clocks using elastic net regression with the *glmnet* package (Friedman et al. 2010) in R v4.3.1 (R Core Team 2023) and evaluated their performance based on median age error and R‐squared.

For feature selection, we identified which classes (age, tissue, sex and genetic population) showed significant differences in DNA methylation patterns. We fitted multivariate linear models with the DNA methylation matrix as the response variable and tissue, sex and population as predictors using the *limma* package (Ritchie et al. 2015). We excluded any CpG sites where DNA methylation differed significantly by class with *p* < 0.001.

## 2 Overview of Epigenetic Clocks

Epigenetic clocks are regression models that estimate an individual's chronological age based on predictable changes to DNA methylation that occur over a lifetime. DNA methylation (DNAm) is an important regulator of gene expression and cellular identity. While many DNA sites and sequences can be methylated, the most frequent and commonly studied form of methylation is the addition of methyl groups on cytosine–guanine sequences (CpG sites; Bestor et al. 2015). Although biologically vital, DNAm is not static over lifetimes. DNAm is both gained and lost with age, resulting in more variable DNAm patterns in older individuals (Meyer and Schumacher 2024). Because most sites are initially highly methylated, this variability generally results in the net loss of DNAm with age (Jones et al. 2015; Jung and Pfeifer 2015). However, some specific highly conserved CpG sites undergo highly predictable changes with chronological age (Horvath 2013; Lu et al. 2023). Epigenetic clocks leverage these predictable, age‐associated changes to estimate chronological age (Hannum et al. 2013; Horvath 2013; Lu et al. 2023).

**FIGURE 2:**
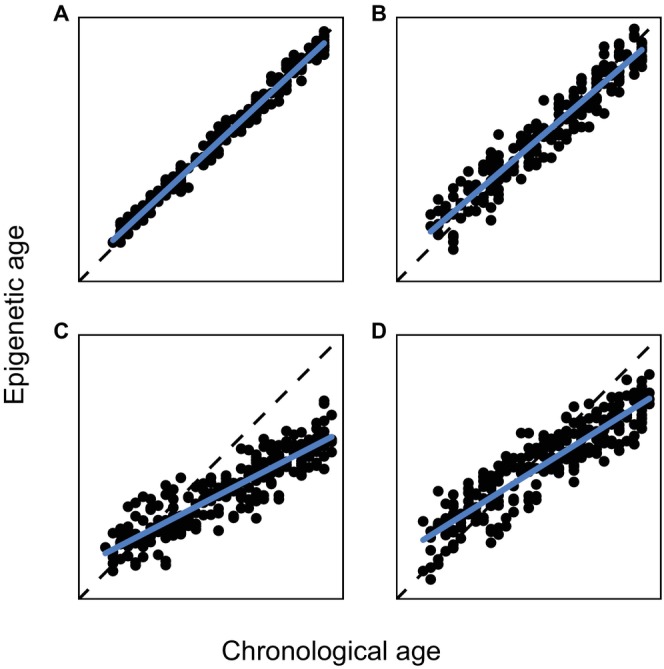
Simulated examples of epigenetic clocks with varying accuracy. The accuracy of epigenetic age estimates can be checked by comparing them to known chronological ages. Black points are observed chronological and predicted epigenetic ages, blue lines are regression lines through the points, and the dotted lines are guides for a 1:1 relationship between chronological and epigenetic age. (A) illustrates a clock with high accuracy. The regression line closely follows the 1:1 line, resulting in low median absolute error (MAE) and high R‐squared and correlation between epigenetic and chronological age. Clock (B) is less accurate, with a higher MAE. Clocks (C) and (D) have a similar correlation and R‐squared, but (D) has a lower MAE, as it better tracks a 1:1 relationship between epigenetic and chronological age.

Epigenetic clock accuracy is typically assessed using the median absolute error (MAE) of the absolute differences between observed chronological and predicted epigenetic ages from a regression model, and either the coefficient of determination (R‐squared) of the linear relationship between epigenetic age and chronological age or Pearson's correlation coefficient (the ‘age correlation’ – Horvath and Raj 2018). A low MAE indicates the clock estimates chronological age with high precision, and R‐squared and age correlation indicate the strength of the linear relationship between epigenetic age and chronological age. Together, a low MAE and high R‐squared or age correlation are characteristics of an accurate clock (Figure 2). When chronological age estimates are reasonably accurate and precise, the residual difference between the chronological and epigenetic ages of an animal, as predicted by the clock, reflects its epigenetic age acceleration (Horvath and Raj 2018), which is a measure of biological age acceleration associated with mortality (Chen et al. 2016; Marioni et al. 2015), disease (Lu et al. 2019) and lifetime stress (Zannas 2019).

Most epigenetic clocks are constructed using elastic net regression (Zou and Hastie 2005). This penalised regression method identifies a small subset of CpG sites, sometimes as few as a dozen out of many thousands, that accurately predict chronological age across a set of DNA samples. Because age‐DNAm relationships are highly correlated among CpG sites, and elastic net regression arbitrarily selects only one of the correlated predictors, the specific CpG sites selected often vary each time the elastic net regression model is fit to the same set of samples (Engebretsen and Bohlin 2019). This means caution should be used in causal interpretations of DNAm related to gene function at specific epigenetic clock sites (Moqri et al. 2023). Still, the resulting age predictions on new samples are generally stable and accurate (Haftorn et al. 2023; Hannum et al. 2013; Horvath 2013).

## 3 Wildlife Applications of Epigenetic Clocks

### 3.1 Potential Applications of Epigenetic Age Estimates in Wildlife Conservation and Management

There are currently two main applications of epigenetic ageing in wildlife studies: accurately estimating the unknown ages of animals to improve information about population age structure and age‐specific vital rates and assessing epigenetic age acceleration as a measure of lifetime stress. While age acceleration is the primary focus of biomedical epigenetic clock research due to its implications for human health, both applications are valuable for wildlife conservation and management.

Other methods for estimating the ages of wildlife are often limited in precision or require invasive sampling (Calvert and Ramsay 1998; Zhang et al. 2024). Morphological biomarkers, such as counts of tooth cementum annuli or aspartic acid racemisation in eye lenses, measure age‐related changes but typically require post‐mortem samples. Additionally, the accuracy of some of these methods varies with age, often providing imprecise age estimates for younger individuals (Garde et al. 2018). Telomere length is another age‐associated molecular marker and a less invasive alternative to morphological approaches, as it can be assessed using tissue samples collected from live animals. However, rates of telomere shortening are highly dependent on the environment and can be inherited across generations, which negatively affects their accuracy when assessing ageing (Le Clercq et al. 2023).

Epigenetic clocks, when trained with accurate chronological ages, are comparatively powerful tools for estimating the ages of individuals whose ages would otherwise be unknown. Less invasive than morphological biomarkers, epigenetic age is also a more accurate reflection of chronological age than telomere length (Le Clercq et al. 2023). The ability to estimate epigenetic age from tissue can increase the number of known aged individuals in a population, which would improve estimates of population growth and survival rates that often coincide with shifts in population age structures (Jackson et al. 2020). By filling these critical gaps, epigenetic clocks will improve our ability to track population dynamics and estimate future trends (Holmes et al. 2007; Hostetter et al. 2021).

Recent research has also identified connections between ecologically relevant environmental stressors and epigenetic age acceleration (Anderson et al. 2021; Newediuk et al. 2024), mirroring biomedical findings and suggesting that epigenetic age acceleration can serve as a useful measure of lifetime stress in wildlife. Unlike traditional wildlife stress biomarkers, such as glucocorticoid hormone levels, which are highly variable and lack a clear reference point for an ‘unstressed’ animal (Romero and Beattie 2022), epigenetic age acceleration is relatively stable and has been consistently associated with stress and health across lifetimes (Lu et al. 2019; Perna et al. 2016; Zannas 2019). However, it should be noted that epigenetic clocks can estimate age acceleration only when applied to known‐age samples, the availability of which can be limited in wildlife studies. We discuss this challenge in Section A.

Importantly, epigenetic acceleration could detect populations experiencing environmental stressors before populations decline, thereby facilitating timely conservation and management interventions. Current metrics for assessing the consequences of stress for populations, such as changes in population dynamics and genetic diversity, are lagging indicators of population health that reflect the cumulative effects of stress following several generations of poor survival and reproductive success. In contrast, epigenetic ageing rates accelerate in response to stress experienced within the lifespan of individual animals, positioning it as a leading indicator to identify populations at risk of future declines.

### 3.2 Recommended Workflow for Designing Accurate Wildlife Epigenetic Clocks

We begin with a discussion of sample collection (Figure 1A), as key decisions made at this stage significantly influence epigenetic clock accuracy. Clock accuracy improves with sample size (Zhang et al. 2019), so clocks should be built with as many samples as possible. Variations in DNAm due to tissue type, sex and genetic ancestry can be addressed during quality control (Figure 1B), pre‐processing (Figure 1C) and clock validation (Figure 1D). However, highly accurate clocks cannot detect the biological differences between chronological and epigenetic age, making them unsuitable for measuring epigenetic age acceleration (Zhang et al. 2019). Measures of epigenetic age acceleration will also be biased when the chronological ages of samples used to train the clock are inaccurate. Moreover, even after pre‐processing and quality control, clocks designed for narrow applications, such as those using samples from and for a single population, may perform poorly when applied to new populations and sample types.

In the following sections, we explore these considerations in more detail, outlining epigenetic clock design decisions related to sample collection, data quality control checks, pre‐processing and clock validation. We provide tailored recommendations for applications of wildlife epigenetic clocks used for estimating the unknown age of individuals and for assessing epigenetic age acceleration.

## 4 Section A – Design Considerations: Sample Selection and Bias

### 4.1 Sampling Challenges in Wildlife Epigenetic Clocks

This section addresses considerations for sample selection when training wildlife epigenetic clocks. In human studies, epigenetic clocks can vary in accuracy when the training set is biased towards one or a few classes of age, sex, tissue, or other factors that influence DNAm (Hannum et al. 2013; McEwen et al. 2020). To mitigate these *class biases* (Box 2), human epigenetic clocks are typically trained on large samples that fully represent the classes to which the clocks will later be applied.

BOX 2 **Class Bias.**

Class biases, which occur when certain categories, such as age, sex, or tissue type, are overrepresented in the data used to train an epigenetic clock, can lower its performance.

**Simulation**

To test the importance of class bias for epigenetic clock performance, we simulated DNA methylation data with a class bias. First, we simulated 500 ß values representing 500 age‐associated CpG sites, in which $\beta_{i}=xy_{i}+\varepsilon$. In our simulated data, $y_{i}$ is a vector of chronological ages from 0 to 30, $\beta_{i}$ represents the proportion of methylation at CpG site i, x is the slope of the relationship between $y_{i}$ and $\beta_{i}$ and ε is normally distributed error (mean = 0, standard deviation = 0.5). We simulated x values for each $\beta_{i}$ from a uniform distribution ranging from −0.1 to 0.1.

We then assigned the simulated samples to one of two types: biased and unbiased. We simulated a weaker association between age and DNA methylation in the biased data by introducing additional error into ε in 5% up to 100% of the CpG sites. We trained two clocks: one using a random sample of 150 each from the biased and unbiased data and another using only samples from the biased data. The second clock represents the case in which a sampling bias might result in a clock designed for one class being applied to predict age in another. We compared the performance of the two clocks using an independent test set of 150 samples from the unbiased class. We fit the clocks using elastic net regression with the *glmnet* package (Friedman et al. 2010) in R v4.3.1 (R Core Team 2023).

**Conclusion**

**FIGURE B1:**
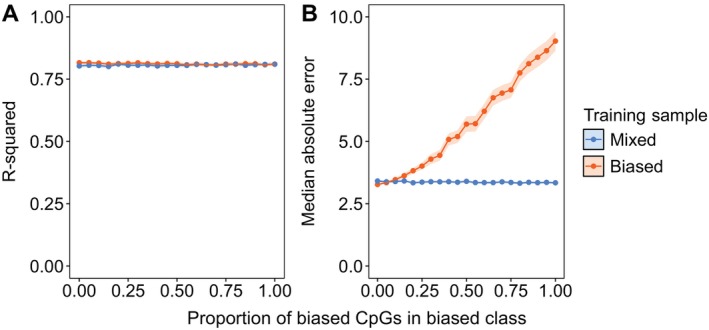
Accuracy of epigenetic clocks trained with two simulated sample types: one without class bias and the other including varying proportions of biased CpG sites (ranging from 0 to 1). The mixed training sample (blue) includes an equal number of samples from both the biased and unbiased classes, while the biased training sample (orange) contains only samples from the biased class. The points and ribbons indicate each accuracy metric's mean and 95% confidence intervals in 100 bootstrapped samples of CpG sites at each proportion.

To ensure accuracy, epigenetic clocks should be trained with all classes of interest. Our results show that class bias does not affect the linear relationship between chronological and epigenetic age (Figure B1A), but it increases the median absolute error (Figure B1B), which grows as the proportion of biased CpG sites increases, suggesting chronological age is either over‐ or underestimated (Figure 2). The median absolute error is minimised when the training set includes samples from both the biased and unbiased classes, as the procedure can select enough age‐related sites to predict age accurately.

In contrast, wildlife sampling is often opportunistic and limited to a single sex, with genetic relationships among the sampled individuals unknown and constraints on the types of tissues that can be collected. Wildlife studies also contend with *age biases*, which arise when sampling is restricted to one or a few age classes. This causes issues when the clock is used on samples collected from individuals whose ages were not represented in the training data (Box 3), exacerbated by non‐linear changes in DNAm with age (Horvath and Raj 2018). For wildlife, non‐linear changes with age also occur during periodic life history stages such as hibernation (Pinho et al. 2022). Additional inaccuracies in wildlife clocks stem from sometimes having to estimate rather than directly measure the chronological ages of sampled individuals (e.g., Mayne et al. 2023; Thompson et al. 2017; Box 3) using ageing techniques that often perform better for some age classes than others (Garde et al. 2018; Hinton et al. 2023), a challenge less important in human studies where chronological ages are usually known.

BOX 3 **Age Bias and Aging Error.**

Training a clock on a narrow chronological age range introduces bias that limits the clock's performance when applied to individuals outside of that chronological age range (Simpkin et al. 2016). The problem is thought to stem from more rapid changes in DNA methylation in some periods of life than others (Alisch et al. 2012), which can be corrected by accounting for the non‐linear relationship between DNA methylation and age (Bernabeu et al. 2023; Haftorn et al. 2023). Another form of age bias arises when the true chronological ages of samples are unknown, introducing ageing error into the chronological ages used for clock training.

**Simulation**

We simulated non‐linear relationships between DNA methylation and chronological age to test the influence of sampling bias on epigenetic clock performance. We simulated 500 ß values, where $\beta_{i}={y_{i}}^{x}+\varepsilon$. In our simulated data, $y_{i}$ is a vector of chronological ages from 0 to 30, $\beta_{i}$ is the proportion of methylation at CpG site i, x is sampled from a normal distribution N(2, 0.35) and ε is normally distributed error *N*(0, 0.8).

Using our simulated data, we trained three clocks using 150 age‐biased samples and tested them on different age groups. First, we trained a clock on 150 individuals aged 0–15 and tested it on 150 samples aged 16–30 to assess how well clocks trained on younger samples performed on older test sets. We then reversed this by training a clock on individuals aged 16–30 and testing it on younger samples aged 0–15. Finally, we trained a clock on samples aged 5–20 and tested it on a broader range of ages (0–30), simulating a common scenario in wildlife research where ‘prime‐age’ individuals are oversampled (Camacho et al. 2017; Smith et al. 1995).

In a second set of simulations, we explored the impact of error in chronological age measurement on clock accuracy. We incrementally introduced ageing error by adjusting the chronological ages of the simulated samples with an error drawn from a random normal distribution with a mean of 0 and a standard deviation ranging from 1 to 5 years. Predicting that a larger training sample might help offset inaccuracy due to ageing error, we fit a series of clocks with ageing error ranging from 1 to 5 years and total sample sizes (i.e., combined training and testing data) ranging from 50 to 1000. We fit the clocks using elastic net regression with the *glmnet* package (Friedman et al. 2010) in R v4.3.1 (R Core Team 2023).

**FIGURE B2:**
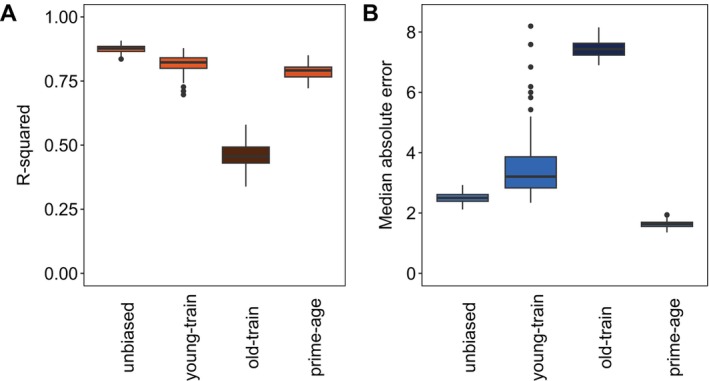
Accuracy of epigenetic clocks, evaluated by R‐squared (A) and median absolute error (B), trained on simulated age‐biased samples and tested on different age groups. From left to right, the clocks are unbiased: trained with the same ages they predict; young‐biased: trained on samples aged 15 years or younger and tested on individuals aged 16–30; old‐biased: trained on samples aged 16–30 and tested on individuals under 15 years; and prime‐aged: trained on samples aged 5–20 and tested on individuals aged 0–30. The colour gradient indicates accuracy. Brighter orange and blue boxes indicate more accurate clocks and darker‐shaded boxes are less accurate.

We found that any form of chronological age inaccuracy reduced clock accuracy. Median absolute error increased when clocks trained on samples of either older or younger individuals were applied to the opposite age class (Figure B2). Both biased clocks also had a lower R‐squared for correlations between chronological and epigenetic ages. Interestingly, the clock trained on prime‐age individuals performed similarly to the unbiased clock, with a slightly lower median absolute error but worse R‐squared.

**FIGURE B3:**
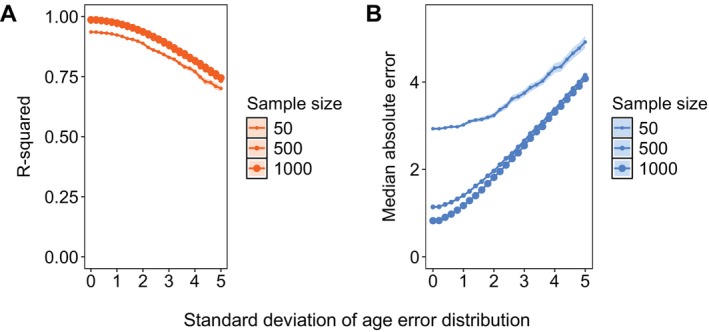
Accuracy of epigenetic clocks with age error and varying sample sizes in predicting chronological age, evaluated by R‐squared (A) and median absolute error (MAE; B). The clocks were trained on simulated data with progressively increasing error (standard deviation) in the training sample ages relative to their true ages and total sample size (training and testing data) ranging from 50 to 1000 samples. The points and ribbons indicate mean accuracy in terms of R‐squared (orange) and MAE (blue) with 95% confidence intervals in 100 bootstrapped samples of CpG sites at each proportion and sample size, with point size reflecting sample size.

Ageing errors also reduced clock accuracy. As we introduced error into sample ages, the median absolute error increased steadily and the R‐squared decreased. Increasing the sample size had little impact on accuracy when the ageing error was high (Figure B3).

**Conclusion**

To increase epigenetic clock accuracy, we recommend avoiding a bias towards exclusively older or younger individuals. Our simulations suggest that accurate clocks can be constructed using samples from prime‐aged individuals, even if sampling regimes cannot capture individuals of very old or very young ages. However, we recommend avoiding under‐sampling young individuals if age bias cannot be avoided. Training clocks with samples from older individuals yielded far worse predictions for young individuals than the reverse, with almost triple the median absolute error of the unbiased clock and an R‐squared lower than 0.5 (Figure B2). This pattern is strikingly similar to findings from many human clocks (Simpkin et al. 2016), suggesting wildlife studies should be particularly cautious of training epigenetic clocks with samples skewed towards older age classes.

Most importantly, ageing error substantially lowered clock accuracy, and the loss of accuracy could not be compensated for by increasing the sample size. When chronological ages were accurate, increasing sample size improved clock accuracy, with the improvement most dramatic between 50 and 500 samples. However, as ageing error increased, increasing the sample size from 50 to 500 had little impact on accuracy (Figure B3). Thus, while large sample sizes of known age individuals can theoretically yield perfectly accurate clocks (Zhang et al. 2019), clock accuracy ultimately depends on training with accurate chronological age data.

Therefore, wildlife studies must recognise the potential limitations of epigenetic clocks trained on class‐biased samples, avoid critical biases related to age, sex, tissue and genetic differences and anticipate the future applications of clocks when collecting samples to build a clock. In the following subsections, we discuss potential causes of reduced clock accuracy due to class and age biases. We then assess variation in the accuracy of clocks that were fit using biased training samples from simulated DNAm data and real DNAm data in which we introduce biases by resampling training and testing data from our polar bear dataset (Box 1). Our analyses demonstrate how age and class biases might affect clock accuracy in wildlife.

### 4.2 Class Biases – Genetic Population Differences in Aging

One of the major class biases in biomedical research is population differences in DNAm patterns associated with ageing. Human population differences arise due to a combination of environmental factors, which account for some between‐population variation in the relationship between DNAm and age, and genetic differences, which also contribute significantly (Carja et al. 2017; Fraser et al. 2012). For example, studies on human twins have shown that genetic differences between individuals can explain up to half of the variation in their epigenetic ageing rates (Jylhävä et al. 2019).

Whether population differences represent an equally important class bias for wildlife epigenetic clocks is uncertain. The Horvath Mammalian Array, the most popular tool used to measure DNAm in non‐human mammals, differs from the analogous human array in that it includes sequences conserved across most mammalian species. This design should minimise bias caused by genetic variation among populations of the same species (Arneson et al. 2022). However, substantial genomic alignment differences to the mammalian methylation array still exist between species (Lu et al. 2023; Zoller and Horvath 2024), suggesting that genetic variation at some sites on the array could also subtly affect clock accuracy between populations.

**FIGURE 3:**
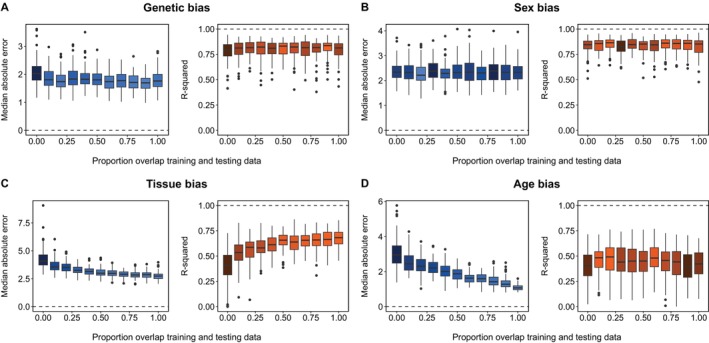
Predictive accuracy of polar bear epigenetic clocks trained with varying levels of class overlap with the testing data, measured by median absolute error (MAE, blue) of epigenetic age relative to chronological age and the R‐squared (orange) of the linear relationship between epigenetic and chronological age. Brighter orange and blue boxes indicate more accurate clocks and darker‐shaded boxes are less accurate. For each overlap proportion, we fit 100 clocks with new training and testing samples, and the resulting accuracy metrics are displayed as boxplots showing the median, interquartile range and outliers. (A) predicts epigenetic age in 30 samples from two western‐Arctic subpopulations (Southern and Northern Beaufort) using 75 samples from the same populations and a genetically distinct central‐Arctic subpopulation (Western Hudson Bay), with overlap proportions ranging from genetically identical (0) to entirely distinct (1). (B) predicts epigenetic age in 30 male samples using 60 samples ranging from entirely female (overlap = 0) to entirely male (overlap = 1), with equal numbers from each subpopulation. (C) predicts epigenetic age in 75 muscle samples from seven subpopulations across the Canadian Arctic, using 100 samples ranging from only muscle (overlap = 1) to blood and skin (overlap = 0). (D) predicts epigenetic age in 30 mature bears (> 5 years) using 45 samples ranging from entirely mature (overlap = 1) to entirely immature (< 5 years, overlap = 0), with equal representation from each subpopulation. The plots indicate that clock performance is most affected by biased tissue types and age groups in the training data and that these biases have a greater impact on the deviation of epigenetic age from chronological age than on the linear relationship between epigenetic and chronological age.

We tested whether population genetic structure affects epigenetic clock performance in polar bears and found minimal evidence of bias (Figure 3A). This suggests age‐related DNAm patterns in this species are largely consistent across populations, despite genetic differences. However, genetic influences on DNAm might vary across species, and many recent species‐specific clocks trained using samples from a single population have not yet been tested on other populations. Cross‐population validations are particularly important because genetic structure often correlates with spatial variation, which could confound relationships between epigenetic ageing rates and environmental variation. Indeed, recent work has already shown different ageing rates between geographically isolated wildlife populations (Cossette et al. 2023).

### 4.3 Class Biases – Sex‐Specific DNA Methylation

In mammals, including humans, females tend to live longer than males (Lemaître et al. 2020). This raises concerns about possible sex‐based differences in epigenetic ageing that could affect the accuracy of epigenetic clocks. The majority of sex‐specific DNAm patterns occur on the sex chromosomes, although some autosomes also exhibit sex‐related differences (Gatev et al. 2021; McCartney et al. 2020). Some clues about the mechanisms behind sex‐related ageing differences come from comparing DNAm between sterilised and unsterilised animals. In these studies, androgen‐sensitive CpG sites in sterilised animals have lower levels of DNAm (Sugrue et al. 2021), and sterilised individuals also age faster epigenetically (Stubbs et al. 2017). In human epigenetic clocks, these biases are well documented; differences in ageing‐related phenotypes between males and females align with distinct DNAm patterns (Grant et al. 2022). To prevent these differences from impacting accuracy, clocks designed for humans and model organisms often exclude markers present on the sex chromosomes (Hannum et al. 2013; Stubbs et al. 2017).

We found no evidence that varying sex ratios in the training dataset affected epigenetic clock accuracy using our polar bear dataset (Figure 3B). This suggests that few sex‐specific sites were selected by the clock. However, sex‐related differences in DNAm could be important in other species, as they have been documented elsewhere (e.g., Czajka et al. 2024; Prado et al. 2021; Robeck et al. 2021).

### 4.4 Class Biases – Tissue‐Specific DNA Methylation

Tissue‐specific differences in ageing rates present a well‐known challenge for building epigenetic clocks (Horvath and Raj 2018; Porter et al. 2021). When sampling live animals, we are often limited to skin, blood and muscle biopsies; however, biobanks, post‐mortem and museum samples and non‐invasive sampling methods (e.g., faeces and hair snags) offer opportunities to sample other tissue types. While the ability to use multiple tissues in clocks will increase the utility of epigenetic clocks, multi‐tissue clocks are also challenging to build and are often less accurate than clocks built with fewer tissue types. In humans, epigenetic clocks trained on a specific tissue tend to be highly accurate for that tissue but less effective for predicting age in other tissues (Porter et al. 2021) as different tissues capture slightly different aspects of ageing (Gibson et al. 2019; McEwen et al. 2020). For example, the human PedBE clock, trained using buccal epithelial cells from children and adolescents aged 0–20 years, remains one of the most accurate human clocks even when applied to older age groups (McEwen et al. 2020). However, its accuracy falls drastically when used to age non‐epithelial tissues (Ibid). In contrast, multi‐tissue clocks can be less accurate but more versatile across different tissues, as they tend not to select tissue‐specific sites (Horvath 2013; Porter et al. 2021).

We found that adding tissue bias in the training set of our polar bear epigenetic clocks significantly reduced clock accuracy (Figure 3C). Clocks designed for other species also demonstrate tissue‐specific differences in DNAm (Robeck et al. 2021; Stubbs et al. 2017). At least in some species, these biases appear to be driven by the elastic net regression algorithm favouring DNAm patterns exclusive to the dominant tissue type in the sample (Robeck et al. 2021).

### 4.5 Age Bias and Age Estimation Bias

Many human clocks are less accurate for ageing young individuals because DNAm changes occur several times faster during this period (Alisch et al. 2012). Rapid changes in DNAm during early life and adolescence are associated with genes related to growth and development that become less active in adulthood (McEwen et al. 2020). Additionally, changes in the cell composition of tissues with age can influence DNAm, as DNAm differs across cell types (Chen et al. 2016; Shireby et al. 2020). When trained on samples with a narrow age range, particularly those from older individuals, clock accuracy declines (Simpkin et al. 2016). For example, the Hannum clock, one of the earliest human clocks, was trained on samples from adults 19 years and older, making it less accurate for adolescents compared to the Horvath clock, which was trained on samples from newborns to older adults (Simpkin et al. 2016). Accounting for non‐linear changes in DNA methylation with age improves the accuracy of epigenetic clocks (Bernabeu et al. 2023; Haftorn et al. 2023).

Chronological age bias is a critical consideration in designing wildlife epigenetic clocks, where sampling methods often favour some age classes over others (Bisi et al. 2011; Camacho et al. 2017; Smith et al. 1995). Thus, some age groups may be underrepresented or absent in many wildlife epigenetic clocks. Using our polar bear data, we found that the MAE increased when we trained clocks with samples from immature individuals and then used those clocks to predict the sample ages of mature individuals (Figure 3D). This suggests that rapid epigenetic ageing rates in young polar bears fail to predict slower rates in adults, a pattern also observed in humans (Alisch et al. 2012). However, clocks trained exclusively on adults, which have slower epigenetic ageing rates than young animals, were even less accurate. When we simulated the non‐linear DNAm patterns that typically occur over a lifetime, we found that training clocks with samples from mature individuals and using those clocks to predict the ages of younger samples resulted in the highest MAE (Box 3).

Moreover, unlike in human studies where chronological ages are typically known, wildlife researchers must often estimate the ages of their samples (e.g., Thompson et al. 2017), introducing further error (Mayne et al. 2023). Methods for estimating wildlife age often rely on body size or changes in the chemical and structural composition of teeth, eyes, baleen, ear plugs and other features as animals age (reviewed in Morris 1972). However, these methods can be inaccurate, leading to either over‐ or underestimation of epigenetic age (Box 3). For example, the accumulation of abnormal proteins in eye lenses is a standard ageing method for bowhead whales ( *Balaena mysticetus* ). This method's low accuracy, in addition to the long lifespan of this species, may explain the poor accuracy of the pan‐mammalian clock in this species (Lu et al. 2023).

### 4.6 Sampling Recommendations for Wildlife Epigenetic Clocks

Based on our simulations, analyses and review of existing epigenetic clocks, it is evident that their accuracy and reliability will be maximised by addressing key sources of bias and sampling either broadly or narrowly, depending on the clock's intended use. We recommend the following approaches to sampling.

#### 4.6.1 Minimise Tissue and Age Biases

Training clocks with accurately aged samples is critical; even large training samples could not compensate for accuracy lost due to ageing error (Box 3). To ensure accuracy, we recommend evenly sampling across ages – particularly ‘prime’ aged individuals that are neither very young nor very old – and either focusing on a single tissue type for clocks designed for use on single tissues or sampling evenly across multiple tissues for broader applications. Our polar bear analysis found tissue and age biases influence clock performance (Figure 3), which is consistent with human studies (Porter et al. 2021). The most accuracy is lost when training samples are skewed towards individuals older than the clock's target population (Box 3). Clock accuracy improves significantly when training samples are skewed towards younger ages, even if the youngest and oldest individuals in a population are not included (Box 3).

Despite being less important than tissue and age biases for polar bears, other class biases, such as population structure and sex differences in DNAm, can also influence clock performance and thus should be considered (Fraser et al. 2012; Grant et al. 2022). If unavoidable, some of these factors can be mitigated using the quality control and pre‐processing methods discussed in sections B and C. Differences in epigenetic ageing rates due to genetic ancestry are particularly relevant for clocks designed to assess epigenetic age acceleration across environments, which could be confounded with genetic variation across environments.

#### 4.6.2 Tailor Sampling to Intended Clock Applications

Our analyses indicate that the most accurate clocks are trained on samples with class characteristics comparable to the test samples (Figure 3; Boxes 2 and 3). We especially recommend aligning the training samples' characteristics with those of anticipated future samples when designing clocks to estimate unknown ages, where a high degree of accuracy is critical.

Conversely, sampling breadth is important for assessing epigenetic age acceleration, as class differences in ageing rates, particularly between populations, could be mistaken for the effects of environmental stressors on epigenetic ageing rates. For example, a clock trained with samples from a single population might predict faster ageing in a different population, either due to genetic differences in age‐associated sites or exposure to distinct stressors. Drawing training samples from both populations should control for the genetic differences. Our simulations suggest that even a small proportion of samples from each class represented in the training sample can improve the clock's predictions across classes (Box 2).

#### 4.6.3 Anticipate Population Dynamics and Sampling Constraints

If future samples will always come from the same tissues, age ranges, populations and sexes, we recommend training the clock with the samples from those classes for maximum accuracy. All our analyses indicated that narrowly focused clocks were the most accurate and other wildlife clock studies have made similar observations (Robeck et al. 2023). However, training on a broader sample range will better capture general age‐related changes in DNA methylation and mitigate future biases from age‐ and class‐specific sites should population demography or sampling methods change. Using data from long‐term research projects to examine past population dynamics will help anticipate these changes to ensure that clocks remain robust to future demographic and genetic shifts.

## 5 Section B – Quality Checks and Data Organisation

Following sample collection, DNAm levels are measured from tissue DNA extractions. The DNA is often bisulfite‐treated to convert non‐methylated cytosines to uracil, which enables their differentiation from methylated nucleotides. Methylation levels at target CpG sites in the bisulfite‐converted DNA are then measured.

The Horvath Mammalian Array is the most widely used platform for measuring DNAm in non‐human mammals. Adapted from earlier microarrays designed for human DNA, the array includes just over 37,000 50‐bp target sites, including conserved CpG sites and their flanking sequences (Arneson et al. 2022). The sites were selected from an alignment of 62 mammal species with the human genome. While not all sites are expected to align to the genome of every mammal species, most genomes tested align to at least half of the sites on the array, and DNAm at a subset of those is expected to change predictably with age (See Section C; Arneson et al. 2022).

In our workflow, we assume readers measured methylation in bisulfite‐converted DNA using the Horvath Mammalian Array. However, alternative approaches are also possible, such as quantifying DNAm in bisulfite‐converted DNA through targeted or whole‐genome next‐generation sequencing (Kurdyukov and Bullock 2016). Regardless of how DNAm is quantified, the considerations we discuss regarding clock design are common to wildlife epigenetic clocks and remain relevant across different sequencing approaches.

In the microarray approach, DNA is extracted from tissue samples and hybridised to the array, stained and imaged. The raw image data are processed to generate individual *beta values*, quantifying the proportion of methylation at each CpG site. These beta values are then normalised to correct for background fluorescence, a component of the technical variation in staining (Triche et al. 2013). The normalised beta values become the input for the elastic net regression model that constitutes the epigenetic clock. R packages, such as *SeSAMe* (Zhou et al. 2018) and *minfi* (Aryee et al. 2014), provide functions for converting the images to raw DNA methylation data and normalising them into beta values. Newediuk et al. (2024) are linked to a well‐structured GitHub project (https://github.com/ljnewediuk/PB_life‐history.git) with detailed R code covering the entire epigenetic clock workflow using *minfi* (Figure 1). A tailored R package, *MammalMethylClock*, also provides detailed workflows and functions for processing data from the Horvath Mammalian Array into normalised betas using *SeSAMe* (Zoller and Horvath 2024).

We recommend several quality‐control checks to prevent technical differences in sample processing from influencing beta values. A key concern is batch effects, which arise because of the structure of DNAm microarrays; each mammal microarray batch consists of four chips with 12 positions each, and variation in DNA hybridisation or staining can introduce systematic variation in fluorescence across chips or chip positions. Batch effects can occur across arrays run on different days, by different staff, or at different facilities. To prevent these artefacts from being mistaken for biological patterns, samples from the same classes should be randomised across chips and batches, even if multiple batches of chips are required. In all cases, batch effects can be assessed and corrected with the *sva* package (Leek et al. 2012) in R.

## 6 Section C – Design Considerations: Data Pre‐Processing Methods

### 6.1 Overview of Pre‐Processing Methods and Wildlife Clock Considerations

After preparing the raw data with normalisation, optional batch correction and other quality control steps, clock performance can still be improved with additional pre‐processing steps before building the epigenetic clock. In this section, we discuss pre‐processing methods that improve performance by reducing the dimensionality of the data used to train the clock. The examples we provide are specific to beta values obtained from the Horvath Mammalian Array, but the same principles of dimensionality reduction are germane to any high‐dimensional DNAm data.

Pre‐processing improves accuracy because DNAm and other high‐throughput data are high‐dimensional, meaning they include many more features (CpG sites) than individuals sampled. This high feature‐to‐sample ratio increases the risk that CpG sites unassociated with age will end up in the epigenetic clock model, introducing unnecessary complexity and reducing clock accuracy. Although the regularisation algorithms used to fit epigenetic clocks mitigate this complexity by penalising the inclusion of uninformative predictors, they do not always eliminate the problem, especially when sample sizes are small. In elastic net regression, the strength of regularisation is controlled by the hyperparameters alpha and lambda, which shrink uninformative sites to zero and may remove them entirely from the model (Kuhn and Johnson 2013). However, high‐dimensional data can still retain correlated features even with regularisation, leading to overfitting. To avoid this problem, many machine learning workflows include feature selection, which involves streamlining the number of features before model fitting. By filtering out uninformative predictors early, feature selection helps prevent overfitting and improves predictive accuracy in new data (Theng and Bhoyar 2024).

Feature selection is likely most beneficial for wildlife epigenetic clocks with small sample sizes. Unlike human epigenetic clocks, often designed using hundreds or even thousands of samples (Fransquet et al. 2019), wildlife clocks often rely on datasets with no more than a few dozen samples (e.g., Czajka et al. 2024; Thompson et al. 2017). This creates an inflated feature‐to‐sample ratio, making dimensionality reduction even more critical. The issue will be compounded for studies quantifying DNAm with whole‐genome sequencing, which results in a feature dataset that is orders of magnitude larger than the Horvath Mammalian Array.

However, a key consideration when incorporating feature selection into epigenetic clock workflows is balancing model simplification with the preservation of predictive information. While feature selection helps to reduce overfitting, it also decreases the number of CpG sites available for epigenetic clock development, potentially excluding important predictive sites if the feature selection is too strict. The importance of retaining predictive sites is evident from studies showing that epigenetic clocks trained with progressively fewer CpG sites can still predict age but with substantially lower accuracy compared to clocks using dozens or hundreds of sites (Haftorn et al. 2023; Li et al. 2022). To find the best balance between minimising the exclusion of important predictive sites and reducing bias from uninformative ones, we applied two pre‐processing approaches – genomic alignment and feature selection – to the polar bear data and assessed their impact on epigenetic clock performance.

### 6.2 Pre‐Processing Methods – Genomic Alignment

An initial approach to reducing the number of features is to align the Horvath Mammalian Array to the genome of the study species before fitting a clock. The array was designed for all eutherian mammals, and while at least half of the sites included on the array are conserved among the 115 species on which it was tested (Arneson et al. 2022), differences in alignments are possible. Genomic alignment, standard practice when using and designing epigenetic clocks (Parsons et al. 2023; Raj et al. 2021; Thompson et al. 2017; Wilkinson et al. 2021; Zoller and Horvath 2024), reduces feature complexity by retaining only CpG sites that align with the genome of the species of interest.

Genomic alignment could exclude as many as 20,000 sites in some species (Arneson et al. 2022), making it an effective method for reducing dimensionality. The use of this approach depends on the availability of a reference genome for the species of interest. Fortunately, many species' reference genome alignments are available on the Mammalian Methylation Consortium's GitHub page at https://github.com/shorvath/MammalianMethylationConsortium/.

### 6.3 Pre‐Processing Methods – Feature Selection

Feature selection methods reduce complexity based on relationships among CpG sites. When sample class characteristics such as sex and genetic population are unknown, features can be retained or excluded from the model using unsupervised methods (Kuhn and Johnson 2013). In variance filtering, for example, CpG sites with the most variation in methylation are retained because sites with low variation are less likely to discriminate among ages (Higgins‐Chen et al. 2022; Sarac et al. 2017; Zhuang et al. 2012). Sites with signals that cluster with other sites are also targets for unsupervised filtering. These sites tend to be more reliable predictors, and retaining them results in accurate and stable clocks (Higgins‐Chen et al. 2022). Related sites can be identified and retained using approaches such as k‐means clustering (Sarac et al. 2017), or clocks can be trained directly on the principal components of multicollinear CpG sites identified with principal components analysis (Higgins‐Chen et al. 2022).

Supervised or semi‐supervised filtering methods select features according to their relationships with explicitly selected class characteristics (Kuhn and Johnson 2013). In epigenetic clocks, the target of supervised feature selection is often age; CpG sites are retained for significant relationships with age (e.g., Li et al. 2022; Zhuang et al. 2012). It is also possible to select features using other target variables. For example, CpG sites can also be excluded for class biases in their DNA methylation–age relationships (e.g., sex – Newediuk et al. 2024). Class bias can be detected with linear models that predict DNA methylation using age and common class‐biased variables such as sex and tissue type (Box 4).

BOX 4 **Feature Selection.**

Feature selection enhances predictive model performance by removing features that lack strong associations with the response variable (Theng and Bhoyar 2024). For epigenetic clocks, supervised feature selection improves accuracy by removing CpG sites with class‐specific relationships between DNA methylation and age. However, excessively reducing the initial pool of CpG sites also limits the features available to model relationships with age, which can reduce model performance (Li et al. 2022).

**Simulation**

We used our simulated class‐biased DNA methylation data, described in Box 2, to test the trade‐off between feature selection and retaining biased features. We assessed the performance impact of retaining versus excluding CpG sites with class‐specific relationships. In the feature selection scenario, we simulated supervised feature selection by sequentially removing the class‐specific CpG sites – from 5% to 95% of the total CpG sites – before fitting the clock. We compared the accuracy of these clocks with those trained using the full set of class‐biased CpG sites.

**Conclusion**

**FIGURE B4:**
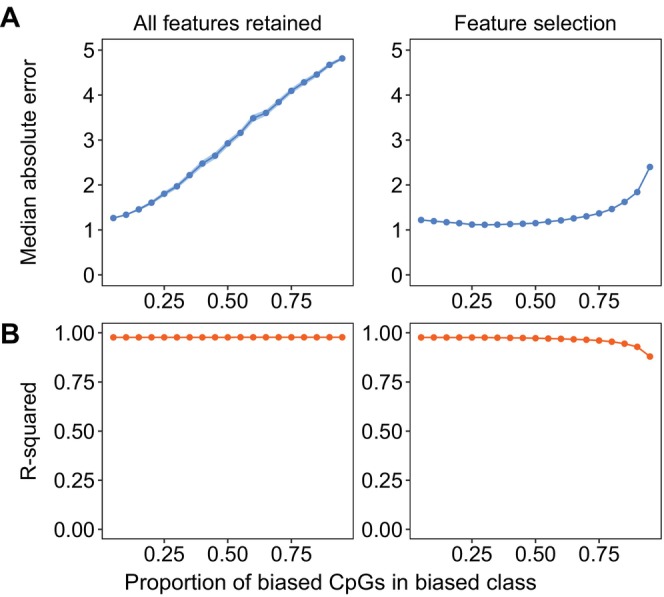
The accuracy of clocks – assessed using (A) median absolute error (MAE) and (B) R‐squared – fit using simulated data as the proportion of biased CpG sites increases in a set of 500 CpG sites. For each proportion, we fit a clock where we retained the biased CpG sites for training and another where we performed feature selection, removing all biased CpG sites before training. The points and ribbons indicate mean accuracy in terms of MAE (blue) and R‐squared (orange) with 95% confidence intervals in 100 bootstrapped samples of CpG sites at each proportion.

Our simulation demonstrates that feature selection for accurate epigenetic clocks requires removing sites that lack any relationship with age while retaining sites important for predicting the age‐DNA methylation relationship. Excluding the class‐biased CpG sites with feature selection kept the median absolute error consistently low relative to clocks in which the class‐biased sites were retained. However, as we removed more CpG sites, the R‐squared declined and the median absolute error increased, indicating that excessively shrinking the initial CpG pool could compromise some aspects of accuracy while improving overall performance (Figure 2). In contrast, while class bias slightly reduced the R‐squared, the removal of class‐biased CpG sites caused an even sharper decline, suggesting excessive feature selection might negatively impact epigenetic clock performance (Figure B4).

**FIGURE 4:**
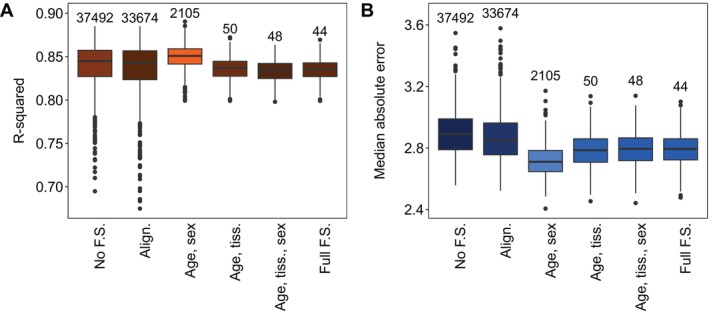
Accuracy, evaluated by R‐squared (A) and median absolute error (B), compared between clocks fit with different feature selection approaches using polar bear methylation data. Each box and whisker represents a different feature selection approach. From left to right, these approaches include no feature selection (No F.S.), sites removed if they did not align to the polar bear genome (Align.), sites removed if they lacked a significant relationship with age in both sexes (Age, sex), sites removed if they lacked a relationship with age in blood, skin and muscle tissues (Age, tiss.), sites removed if they lacked a significant relationship with age in all tissues and both sexes (Age, tiss., sex) and sites removed if they lacked a relationship with age in all tissues and both sexes and did not align to the polar bear genome (Full F.S.). In each approach, we fit 500 clocks by selecting 319 individuals for training sampled evenly across subpopulations, ages 0–30 years, sexes and all tissue types, then applied them to predict the ages of the remaining 250 individuals. Numbers above the boxes and whiskers denote the number of initial sites retained for fitting the clock. Brighter orange and blue boxes indicate more accurate clocks and darker colours are less accurate.

We found that removing biased CpG sites from our polar bear clocks through supervised feature selection – retaining those features related to age but not dependent on sex or tissue type – improved accuracy relative to genomic alignment alone (Figure 4). This is likely because feature selection eliminated at least 94% of sites on the array, substantially more than the 10% (3818 sites) eliminated because they did not align with the polar bear genome.

However, excluding too many sites with feature selection compromised accuracy. For example, removing 35,387 sex‐specific sites and those without a strong relationship with age left 2105 sites to create the clock, which reduced the MAE compared to clocks without feature selection (Figure 4B). Additionally, removing tissue‐biased sites and those that did not align with the polar bear genome resulted in the elimination of 37,448 sites, leaving only 44 sites to create the clock, which caused a sharp decline in R‐squared (Figure 4A).

Our feature selection scenarios highlight a fundamental consideration in building epigenetic clocks: reducing feature complexity improves accuracy only up to a point. Our feature selection simulations showed that removing class‐biased sites reduced clock MAE and maintained a high R‐squared until the number of removed sites reached a threshold, beyond which accuracy declined sharply (Box 4). For our polar bear clocks, this threshold occurred somewhere between the removal of 35,387 and 37,442 CpG sites, representing 94.4% and 99.9% of sites on the Horvath Mammalian Array. Excessive feature selection may also compromise estimates of age acceleration, as too few sites will remain to capture the biologically meaningful variation in DNAm caused by lifetime stress.

Nevertheless, feature selection is important for wildlife epigenetic clocks, particularly those using whole‐genome bisulfite sequencing, where small sample sizes and large genomes inflate the feature‐to‐sample ratio and reduce accuracy. Site removal can also be fine‐tuned during model fitting by setting the regularisation hyperparameters alpha and lambda closer to their maximum values, which will result in stricter removal of uninformative sites.

## 7 Section D – Design Considerations: Validation Approaches

Evaluating epigenetic clock accuracy is a critical step in their development because it ensures accuracy when applied to new samples. The gold standard involves validating the clock on a hold‐out dataset not used for clock training. This method, widely used in human epigenetic clock studies with large sample sizes (e.g., Hannum et al. 2013; Horvath 2013; McEwen et al. 2020), leaves enough samples to create an accurate clock while avoiding inflated accuracy estimates caused by overfitting the training dataset (Hastie et al. 2009). However, in wildlife studies, small sample sizes make it difficult to reserve a substantial hold‐out set for validation without severely limiting the data available for clock training. Validation strategies for wildlife studies must, therefore, maximise true accuracy while avoiding its overestimation. This section discusses the benefits of different validation approaches, comparing them using our polar bear data.

There are three primary approaches for selecting a validation set to estimate the accuracy of predictive models, including epigenetic clocks. In addition to setting aside a distinct hold‐out set or using the same dataset for both training and validation, validation can be performed on a series of smaller subsets of the training data, with errors averaged across subsets sampled from the training data – a method known as cross‐validation.

Cross‐validation approaches differ by the size of equally sized subsets or folds, *k*. In *k‐fold cross‐validation*, a fold of size *k* is used for training, while the remaining *k‐1* folds are used for testing. *Leave‐one‐out (LOO) cross‐validation* is a special case of *k‐fold cross‐validation*, in which each fold contains only a single observation. In the context of epigenetic clock models, the single observation can also be a single grouping of individuals. For example, the universal clock for mammals was validated using leave‐one‐species‐out cross‐validation, in which the clock, trained on all but a single species, was tested on each excluded species in turn (Lu et al. 2023). In species‐specific clocks, the group could be population, sex, or tissue, with the remaining groups used for testing.

The small sample sizes typical of wildlife studies often make cross‐validation the only practical option for epigenetic clock validation. Indeed, most wildlife clocks published since the release of the Horvath Mammalian Array – including the universal clock for mammals – were validated using LOO cross‐validation (e.g., Parsons et al. 2023; Prado et al. 2021; Raj et al. 2021; Robeck et al. 2021). LOO cross‐validation estimates true test error well because it uses nearly all the data (*n*–1) for training while iterating systematically through the testing data (James et al. 2013).

Using our polar bear dataset, we evaluated the accuracy of epigenetic clocks validated through LOO cross‐validation and compared it to validation on an independent hold‐out set. First, we randomly selected 400 polar bear samples. Within this subset, we sampled 250 for training the clock. We retained the remaining 150 samples as a hold‐out set and also validated the clock by performing LOO cross‐validation on all 400 subsetted samples. We repeated this process 100 times.

**FIGURE 5:**
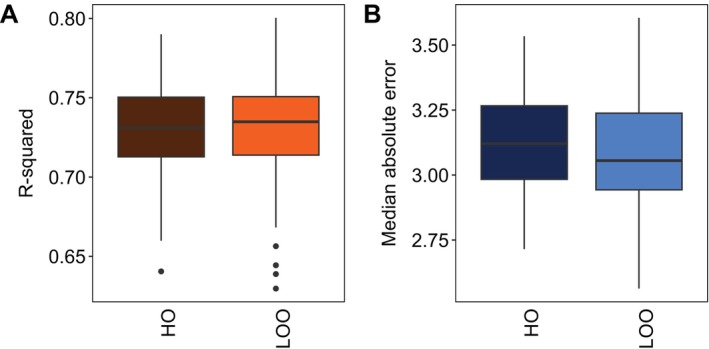
Accuracy, evaluated by R‐squared (A) and median absolute error (B), compared between polar bear clocks validated using leave‐one‐out cross‐validation (LOO) versus an independent hold‐out set (HO). Bright orange and blue boxes represent higher accuracy, while darker colours represent lower accuracy.

Our results suggest LOO cross‐validation, known to approximate the accuracy of hold‐out clocks in other machine learning applications (Hastie et al. 2009), also does so for epigenetic clocks. We found no difference in either the R‐squared (Figure 5A) or MAE (Figure 5B) between clocks validated with hold‐out data and LOO cross‐validation (Figure 5B), indicating both approaches capture true accuracy equally well. Importantly, this suggests that wildlife clock developers may be justified in using their full set of available samples to maximise clock accuracy while still reliably assessing the clock's predictive performance when applied to new samples.

## 8 Conclusions

Epigenetic clocks have the potential to fill critical data gaps in wildlife conservation and management. However, challenges associated with collecting wildlife DNA samples that can negatively affect the accuracy of epigenetic clocks have been largely unexplored. The absence of a standardised workflow for developing wildlife epigenetic clocks also hampers their widespread use. To address these issues and encourage their development, we provided a detailed workflow for developing epigenetic clocks geared towards wildlife research (Figure 1), encompassing sample selection, quality control, feature pre‐selection and validation. We demonstrated our recommended workflow using simulations and data from polar bears across the Canadian Arctic, equipping practitioners with the tools and knowledge needed to design and develop accurate epigenetic clocks.

Through our polar bear analyses and simulations, we showed that thoughtful sampling, feature selection and validation can produce accurate epigenetic clocks for wildlife, even with small sample sizes. Among our most important recommendations is to plan ahead of clock development, as identifying target populations, tissues, age ranges and sexes in advance enables the design of wildlife clocks tailored to specific applications. Narrowly focused clocks are often the most accurate, except when applied to a broader range of samples than those on which they were trained. Clock accuracy can also be enhanced by reserving fewer sites for testing or using all available samples for training. While maximising accuracy is particularly important for clocks used to estimate unknown ages, perfectly accurate clocks cannot measure epigenetic ageing rates, emphasising the need to clarify the clock's intended purpose from the outset.

Finally, we note that identifying and remedying the sources of error and bias we explored in this review requires detailed knowledge of the study system (e.g., genetic structure, age structure). These issues should be considered at the earliest planning stages, particularly when bioinformatics work is outsourced to service providers without knowledge of study systems and potential sample biases. With planning, epigenetic clocks can provide highly accurate age data for wildlife conservation and management.

## Author Contributions

L.N., M.J.J., C.J.G. and E.S.R. conceived of the study. A.M.B. and A.R.C. contributed data. M.J.J. contributed lab resources. L.N. analysed the data and wrote the manuscript. All authors provided feedback on the manuscript.

## Disclosure

All collaborators contributing community data for this study were offered authorship. The results of this work are shared with the local communities through these contributors.

## Conflicts of Interest

The authors declare no conflicts of interest.

## Data Availability

All data and code are available from https://github.com/ljnewediuk/how_to_clocks.git. The data are also available on Dryad at https://doi.org/10.5061/dryad.rxwdbrvmw.
